# Formation of Spatially Discordant Alternans Due to Fluctuations and Diffusion of Calcium

**DOI:** 10.1371/journal.pone.0085365

**Published:** 2013-12-31

**Authors:** Daisuke Sato, Donald M. Bers, Yohannes Shiferaw

**Affiliations:** 1 Department of Pharmacology, University of California Davis, Davis, California, United States of America; 2 Department of Physics and Astronomy, California State University Northridge, Northridge, California, United States of America; University of Minnesota, United States of America

## Abstract

Spatially discordant alternans (SDA) of action potential duration (APD) is a phenomenon where different regions of cardiac tissue exhibit an alternating sequence of APD that are out-of-phase. SDA is arrhythmogenic since it can induce spatial heterogeneity of refractoriness, which can cause wavebreak and reentry. However, the underlying mechanisms for the formation of SDA are not completely understood. In this paper, we present a novel mechanism for the formation of SDA in the case where the cellular instability leading to alternans is caused by intracellular calcium (Ca) cycling, and where Ca transient and APD alternans are electromechanically concordant. In particular, we show that SDA is formed when rapidly paced cardiac tissue develops alternans over many beats due to Ca accumulation in the sarcoplasmic reticulum (SR). The mechanism presented here relies on the observation that Ca cycling fluctuations dictate Ca alternans phase since the amplitude of Ca alternans is small during the early stages of pacing. Thus, different regions of a cardiac myocyte will typically develop Ca alternans which are opposite in phase at the early stages of pacing. These subcellular patterns then gradually coarsen due to interactions with membrane voltage to form steady state SDA of voltage and Ca on the tissue scale. This mechanism for SDA is distinct from well-known mechanisms that rely on conduction velocity restitution, and a Turing-like mechanism known to apply only in the case where APD and Ca alternans are electromechanically discordant. Furthermore, we argue that this mechanism is robust, and is likely to underlie a wide range of experimentally observed patterns of SDA.

## Introduction

When cardiac tissue is rapidly paced, the action potential duration (APD) can alternate in a LSLS sequence of long (L) and short (S) APDs referred to as alternans[1-3]. This phenomenon can become severe when the heart is in pathological conditions, and often leads to spatially discordant alternans (SDA) where different regions of tissue alternate in a sequence that is out-of-phase i.e. one region alternates in a LSLS pattern while a nearby region exhibits SLSL [4,5]. It has been argued that SDA is arrhythmogenic since it will lead to the formation of large gradients of refractoriness that can cause wave break and reentry [4,6,7]. However, the mechanisms for the formation of SDA are not completely understood. This is partly because the formation of alternans at the single cell level can occur due to a variety of distinct mechanisms [8], and it is not clear how this will influence the dynamics of SDA formation on the tissue scale. In particular, it has been shown that alternans at the cellular level can be induced by an instability of membrane voltage, which is due to steep APD restitution caused by the nonlinear kinetics of ion channels of membrane currents, or due to Ca cycling, which is due to a steep relation between the Ca release from the sarcoplasmic reticulum (SR) and the SR Ca load [9]. Moreover, the coupling between voltage and Ca can be positive when the sodium-Ca exchanger dominates or negative when the L-type Ca current dominates. To date, two mechanisms for SDA have been identified; one is a purely voltage mechanisms that relies on the non-uniform time of arrival of paced excitations, which is caused by a steep conduction velocity (CV) restitution [10-13], and the other occurs due to a Turing-like pattern forming instability which arises when APD and Ca alternans are negatively coupled (i.e. a larger Ca transient will tend to shorten the APD) and electromechanically discordant [14,15]. 

In this study, we present a novel mechanism for SDA in cardiac tissue that applies when alternans is caused by an instability of Ca cycling and where Ca and APD alternans are electromechanically concordant. This scenario is physiologically relevant since Ca cycling has been identified as a key factor in the induction of various cardiac arrhythmias [16-20]. Furthermore, it is well documented that Ca cycling can drive alternans under conditions of rapid pacing which leads to Ca overload [9,21-23]. This mechanism applies in the case where Ca alternans development occurs slowly, over several seconds to minutes of pacing, as Ca accumulates in the SR in response to an abrupt decrease in CL. We show that this slow development of Ca alternans amplitude leads to spatially out-of-phase Ca alternans, since Ca diffusion is not sufficient to synchronize alternans phase across the length scale of a cell. Thus, subcellular patterns of out-of-phase alternans will form with a length scale that is set by the competition between the underlying fluctuations of Ca cycling, and the smoothing effect of subcellular Ca diffusion. These spatially discordant patterns then gradually coarsen as the Ca transient alternans induce tissue scale APD alternans, which tends to synchronize the subcellular Ca release patterns. However, this synchronization only occurs over a length scale set by the electrotonic coupling in tissue, and therefore, regions that are sufficiently far apart will tend to develop APD alternans that are out-of-phase. Thus, SDA will occur at steady state with a length scale that is dictated by the electrotonic coupling in cardiac tissue. Furthermore, we argue that this mechanism is relevant to a wide variety of physiological conditions, and is likely to explain experimentally observed SDA patterns in the case when the underlying dynamical instability is caused by unstable Ca cycling.

## Materials and Methods

### A multi-scale model of voltage and Ca in cardiac tissue

**Figure 1:**
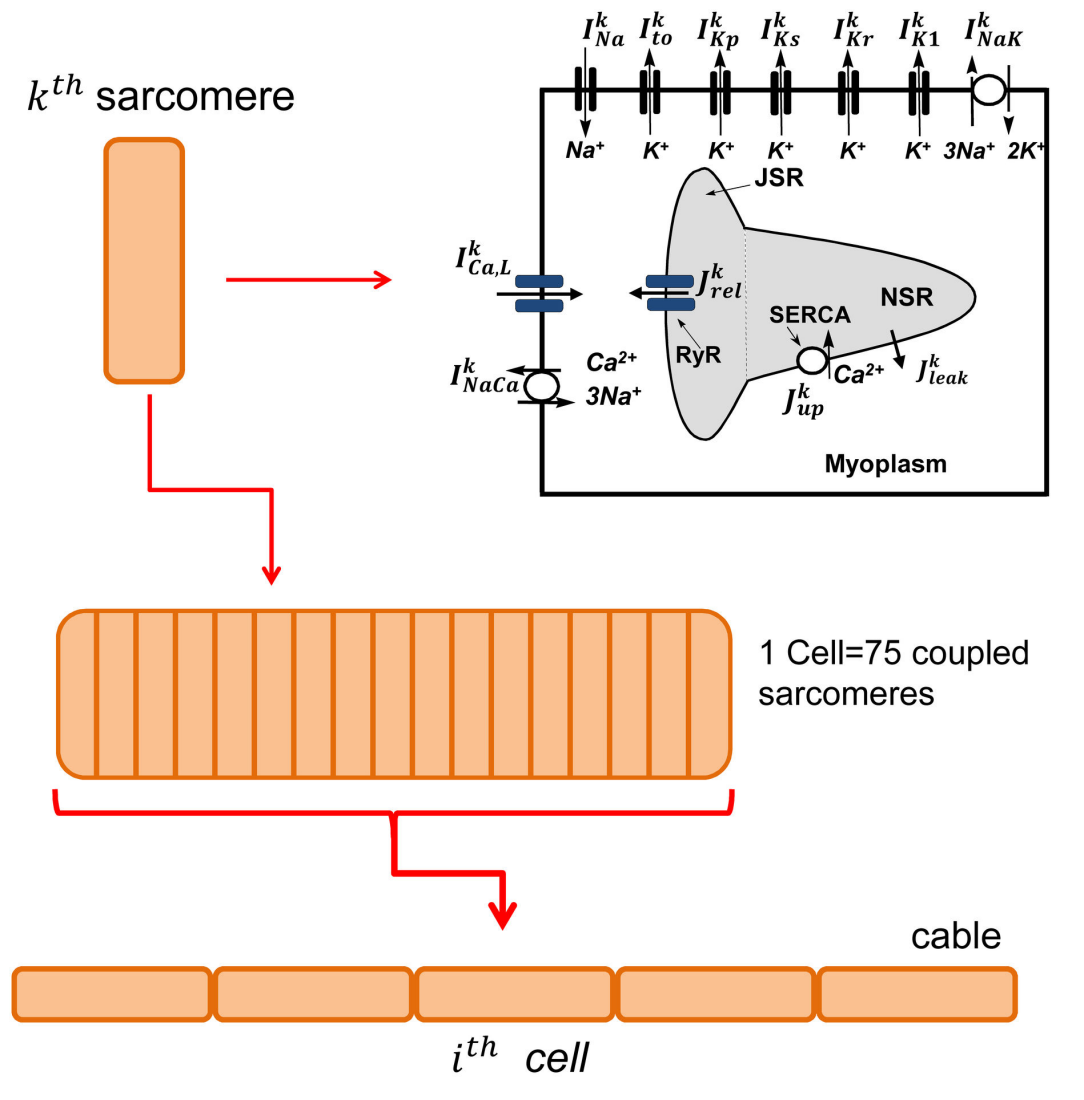
Schematic illustration of the multi-scale computational model. Each cell consists of 75 diffusively coupled sarcomeres. Ca concentrations within each sarcomere are modeled using phenomenological equations describing Ca cycling. Ca sensitive ion channels are labeled with a superscript *k*, while non Ca sensitive channels are the same for all sarcomeres. Neighboring cells are coupled via gap junctions in tissue.

We have developed a multi-scale computational framework to model voltage and Ca at the sarcomere, whole cell, and tissue scales. Following Ref. [24,25] we model a cardiac cell as a collection of 75 sarcomeres that are coupled via Ca diffusion through the cytosol and the network SR (NSR). Each sarcomere is divided into four (cytosol, submembrane, network SR, and junctional SR) subcellular compartments, as illustrated in Figure 1, which contain the appropriate Ca fluxes from the RyR, SERCA, LCC (*I*_*Ca*_) and sodium-calcium exchanger (*I*_*NaCa*_). Ionic currents are modeled following Sato et al. [14] who coupled an established ionic model [26] with the Ca cycling model of Shiferaw et al[27]. As described in [8] this phenomenological model can be conveniently used to explore the scenario in which alternans are due to an instability of Ca cycling caused by a steep SR release-load relationship, or alternatively via a steep APD restitution curve. The spatial distribution of Ca is modeled by computing Ca concentrations within each compartment using ordinary differential equations (ODEs) describing the spatially averaged dynamics within a sarcomere. To model the membrane voltage (*V*_*m*_) we assume that voltage is spatially uniform so that all sarcomeres within a cell share the same voltage time course. This assumption is valid since *V*_*m*_ diffuses across a cell (~100 µm) in roughly (~0.1 ms), which is much faster than all other relevant time scales in the system. In order to understand the relationship of alternans between cells, we also simulate a multi-cell system of a few (2-5) cells, as a collection of groups of 75 sarcomeres that are electrotonically coupled. Again, since voltage diffusion is fast on this length scale we assume that all sarcomeres share the same voltage time course. In this study, we have set Ca diffusion between cells to zero, since experimental studies have shown that Ca diffusion across gap junctions is small [28]. 

To model voltage in one and two-dimensional tissue we apply the cable equation:

$$\frac{\partial V}{\partial t}=-\frac{I_{ion}}{C_{m}}+D_{V}\left(\frac{\partial^{2}V}{\partial x^{2}}+\frac{\partial^{2}V}{\partial y^{2}}\right)$$ (1)

where *C*_*m*_=1 µF/cm^2^ is the membrane capacitance, *D*_*V*_=10^-3^ cm^2^/ms is the effective voltage diffusion coefficient, and where *I*_*ion*_ is the total transmembrane current. The cable equation as integrated using an operator splitting approach [29], with space step Δ*x*=0.015 cm, and with a variable time step in the range *dt*=0.01~0.1 ms. To model the dynamics of Ca and voltage in tissue we assign each lattice site a voltage *V*_*i,j*_, which is coupled to Ca dynamics on the sarcomere scale. Here, Ca in each cell is labeled with an index *k*=[1,2,…,*M*] where *M* is the number of sarcomeres. The voltage time course is then modeled by computing *I*_*ion*_ at each cell as a summation of contributions from individual sarcomeres. The ionic current at each cell is then given by

$$I_{ion}^{i,j}=I_{Na}^{i,j}+I_{K}^{i,j}+\sum_{k=1}^{M}\left(I_{NaCa}^{i,j,k}+I_{Ca}^{i,j,k}\right)$$ (2)

where $I_{Na}^{i,j}$ and $I_{K}^{i,j}$ are the total sodium and potassium currents at the cell on lattice site *i,j*, and where the contributions of the sodium-Ca exchanger and the L-type Ca current are summed over all *M*=75 sarcomeres in that cell. Note here that we have assumed that each sarcomere is driven by the same non Ca sensitive ion currents. We pace the cell to steady state at the slow rate (CL=600ms) and use the steady state values as initial conditions.

### Spatiotemporal dynamics

To keep track of the spatial distribution of Ca transient alternans at the sarcomere scale, we define amplitudes of Ca transient alternans and APD alternans as follows. The peak of the Ca transient at a sarcomere will be denoted as *c*_*n*_ and the amplitude of Ca transient alternans is computed as 

$$\Delta c_{n}={\left(-1\right)}^{n}\frac{\left(c_{n}-c_{n-1}\right)}{2}$$ (3)

where the factor (-1)^*n*^ is included so that positive and negative values of Δ*c*_*n*_ correspond to opposite alternans phase. Likewise, the amplitude of APD alternans is measured using the quantity

$$\Delta a_{n}={\left(-1\right)}^{n}\frac{\left(a_{n}-a_{n-1}\right)}{2}$$ (4)

where *a*_*n*_ is the APD measured at beat *n*. To keep track of the spatiotemporal dynamics of alternans in 2D tissue we compute the alternans amplitude at cell *i,j* denoted as Δ*a*_*n*_(*i,j*) and also Δ*c*_*n*_(*i,,j,k*), where *k* denotes the saromere index. With these definitions, the nodes separating spatially out-of-phase regions of Ca transient and APD alternans are located at locations Δ*a*_*n*_(*i,j*)=0 and Δ*c*_*n*_(*i,,j,k*)=0 so that nodes of Ca alternans are resolved at a single sarcomere scale, while nodes of APD alternans on the cellular scale.

### Stochastic fluctuations

In this study, we model the beat-to-beat fluctuations of Ca cycling dynamics at the sarcomere scale. These fluctuations can occur due to a multitude of factors such as ion channel stochasticity, fluctuations in Ca concentrations within subcellular compartments, and also on the stochastic nature of Ca spark recruitment. This effect is crucial in our study since Ca cycling fluctuations influence the development of alternans phase at the early stages of pacing when alternans amplitude is small. To model fluctuations in Ca cycling we follow Sato et al [14]. and account for the finite number of SERCA pumps in the cell by formulating an uptake current described by a Langevin equation with a noise term that depends explicitly on the number of pumps within a sarcomere. Details of the current dynamics and noise formulation are given in the online data supplement. The C++ code is available upon request. In this study, we chose the amplitude of the noise to be about 10% of the average uptake current. We stress that all results presented here are sensitive to the presence of fluctuations but are independent of the specific source of those fluctuations.

### Cell model parameters

In this study we model *V*_*m*_ and Ca dynamics using an established ionic model [24] which can be tuned to reproduce experimentally measured features of Ca transient alternans. In this model, alternans at the cellular level can be induced by an instability of Ca cycling that is due to a steep dependence of the SR Ca release as a function of SR load, which occurs under Ca overload conditions. Here, the gain of the Ca release is adjusted so that Ca alternans occurs at steady state at fast rates (CL<300 ms). To induce this instability we simply pace our cardiac cell to steady state at slow rates (CL=600 ms), and then make a transition, over one beat, to a rapid pacing rate (CL=300 ms). Since Ca accumulation is slow, the steady state Ca concentration occurs after roughly 100 beats. Also, model parameters are adjusted so that fully developed alternans are always electromechanically concordant, so that a large (small) Ca transient corresponds to a long (short) APD. This choice is motivated by numerous simultaneous voltage and Ca (or contraction) measurements, under a wide variety of conditions and species, which show an electromechanically concordant relationship[7,9,30-32]. This parameter regime corresponds to the case of "positive coupling" [8], which occurs since a large Ca release will, due primarily to the sodium-Ca exchanger, tend to prolong the APD. 

## Results

### Formation of alternans at the single sarcomere level

**Figure 2:**
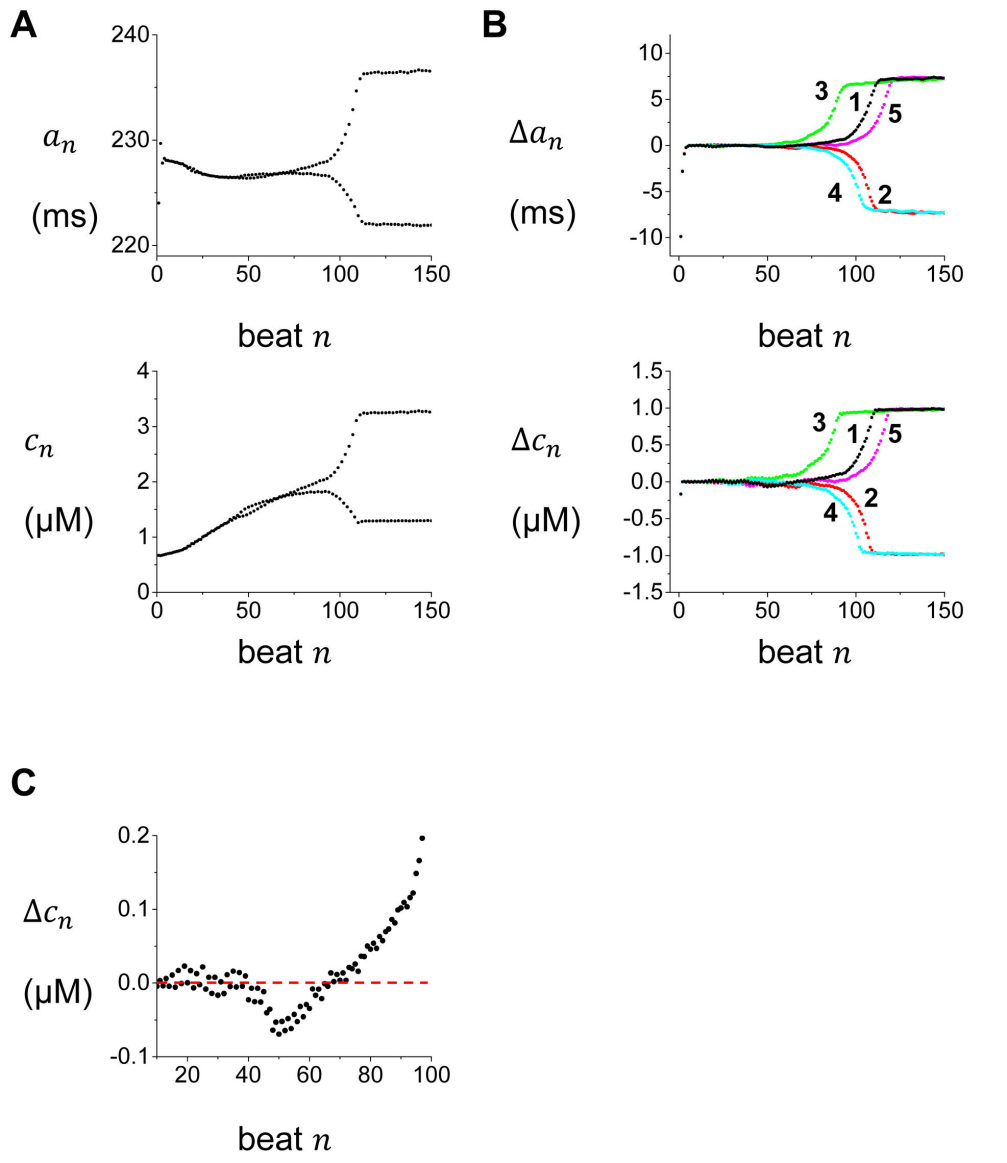
APD and peak CaT during development of alternans after a change in CL. (A) APD (top) and peak Ca transient (bottom) vs beat number *n*. (B) The amplitude of APD (Δ*a*_*n*_) and Ca transient (Δ*c*_*n*_) alternans amplitude vs. beat number for five independent simulations with identical parameters and initial conditions. Differences between each simulation run are due only to the Ca cycling fluctuations present. (C) The amplitude (Δ*c*_*n*_) of Ca transient alternans vs beat number *n* at the early stages of pacing.

In order to understand the spatiotemporal dynamics of Ca and APD alternans on a tissue scale, it is first necessary to characterize alternans development at the level of a single sarcomere. Using our computational model we induce alternans by pacing a sarcomere at a rate for which the beat-to-beat Ca release is periodic (PCL=600 ms), and then making a sudden change in cycle length to a rate where the steady state response displays alternans (PCL=300ms). In Figure 2A we plot both the peak of the Ca transient and APD at beat *n*, which we denote as *c*_*n*_ and *a*_*n*_, respectively. We observe that Ca transient alternans amplitude grows slowly and is fully developed after roughly 100 beats. We note here that near beat 50 alternans amplitude grows but then dissipates due to Ca cycling fluctuations. However, by beat 70 the amplitude of Ca alternans grows large enough such that fluctuations are not sufficient to reverse phase. As Ca accumulation increases further the alternans amplitude increases to its steady state value. In Figure 2B we plot the evolution of the amplitude of APD and Ca alternans for five independent simulations. In these simulations, all parameters are kept the same so that only the fluctuations due to the finite number of SERCA pumps account for the distinct time evolution. These results indicate that while the development of alternans is similar in all cases, the sign of the final phase of alternans is random, with an equal probability of being positive or negative. To further investigate the nature of this stochastic process in Figure 2C we plot the beat-to-beat evolution of Ca alternans amplitude. Note, that during this time interval, alternans amplitude is small and the stochastic noise from the finite number of SERCA pumps is sufficient to perturb alternans phase from one beat to the next. In other words, phase can be reversed such as lslslsslsl. However, following the 70th paced beat the Ca alternans amplitude grows to a magnitude such that fluctuations are too small to influence the phase of alternans. In other words, the sequence here is always lsls or slsl and cannot be flipped due to Ca cycling fluctuations. Therefore, the final steady state alternans amplitude is critically sensitive to the stochastic perturbations at earlier beats in the pacing sequence. Later, we will show that this underlying sensitivity to Ca cycling fluctuations is a critical factor to form SDA on the whole cell and tissue scale. 

### Alternans formation and drift within a cardiac cell

**Figure 3:**
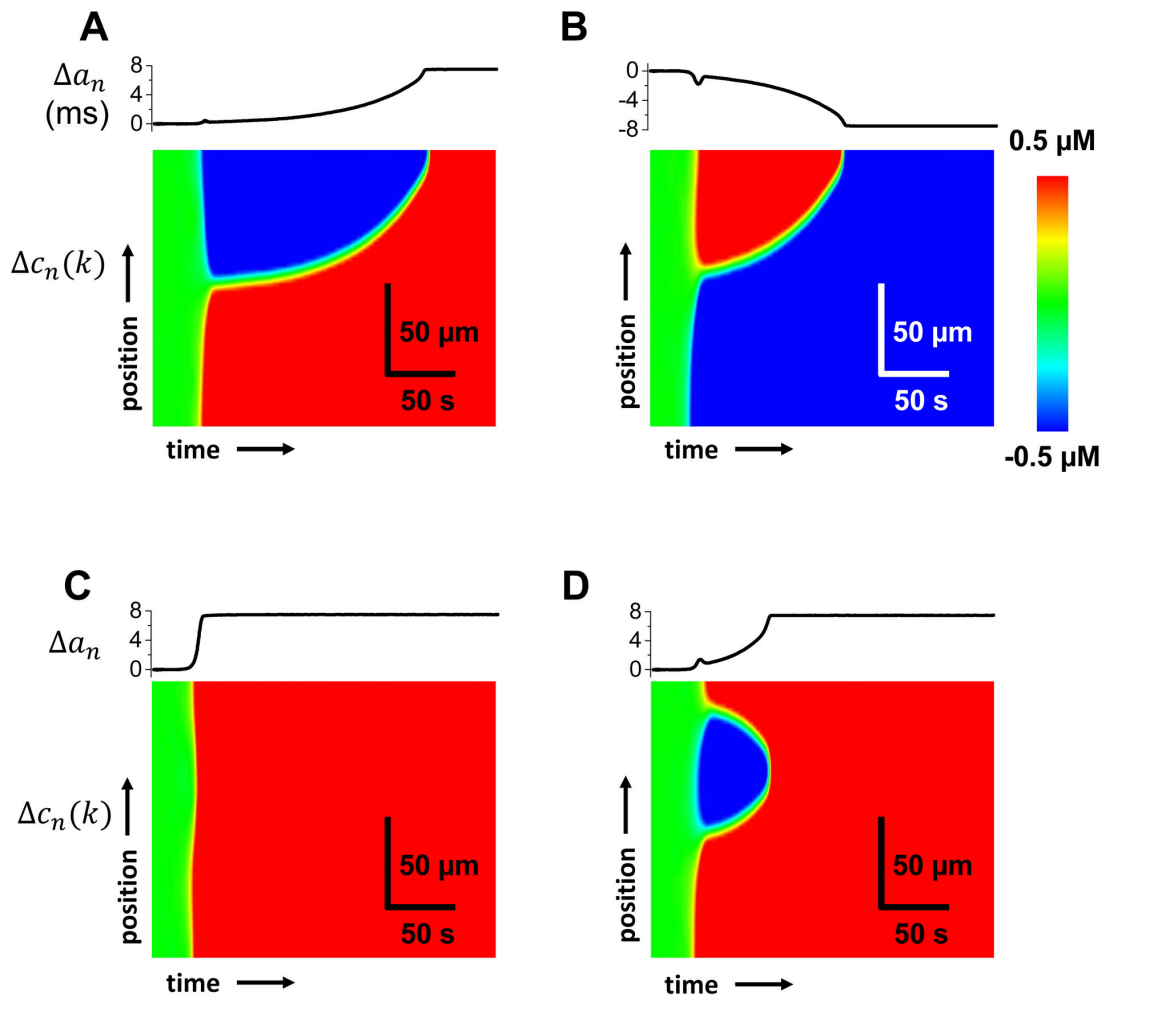
Subcellular Ca dynamics in a single cell during development of alternans. (A-D) Four independent simulations with the same parameters and initial conditions are shown. Each panel has the amplitude of APD alternans (top) and space-time plot of the amplitude of Ca transient alternans (bottom).

We have applied our pacing protocol to simulate the spatiotemporal evolution of Ca and APD alternans within a single cardiac cell of 75 coupled sarcomeres. On this scale we keep track of the spatiotemporal distribution of Ca transient amplitude at a sarcomere *k*, denoted as Δ*c*_*n*_(*k*), along with the APD alternans amplitude Δ*a*_*n*_. In Figure 3A-D we show the space-time plot of the amplitude of Ca alternans in response to a sudden change in *PCL*at time *t*=0. Here, each image corresponds to an independent simulation with identical parameters, so that only random fluctuations account for the distinct time evolution. We observe that in cases A,B and D the spatial distribution of Ca alternans is heterogeneous with different regions of the cell exhibiting alternans with opposite phase separated by a node, while in case C no node was formed. The color scheme we apply shows a lsls pattern as red, and a slsl pattern as blue, with a green line demarcating a node (Δc=0) which separates the two phases. We note that discordant patterns are formed early, after the first 50s of pacing, and that the nodes drift slowly and extinguish by colliding with the cell boundaries (A,B), or with a node moving in the opposite direction (D). Therefore, in all cases the steady state pattern of alternans is spatially synchronized, with a phase that can be lsls or slsl with equal likelihood. 

### Coupling between APD and subcellular Ca alternans

**Figure 4:**
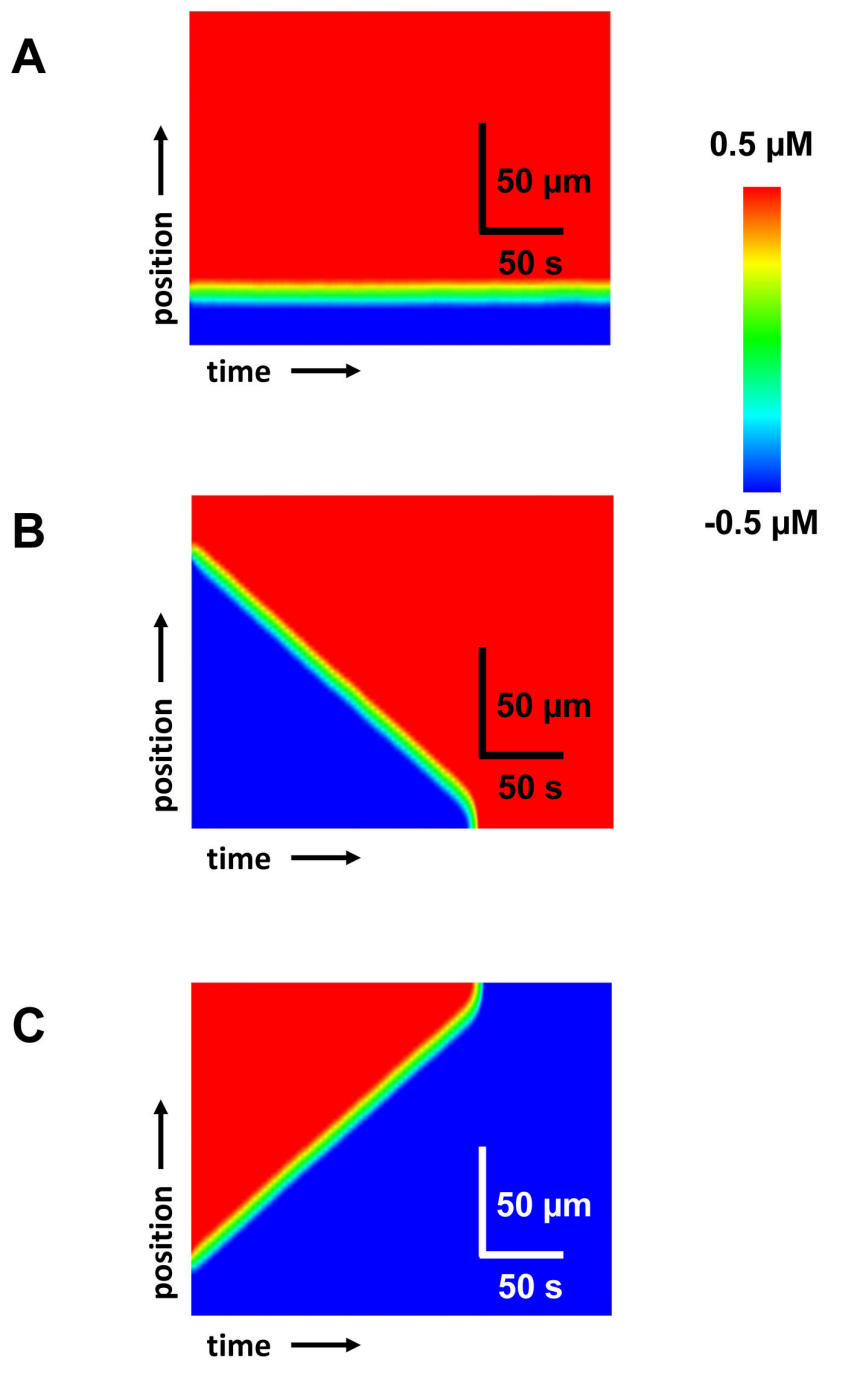
Subcellular Ca dynamics when a cell is paced with an AP clamp waveform. (A) Periodic AP clamp. (B-C) AP clamp alternating in a LSLS and SLSL sequence respectively. AP clamp is taken from a recording of a single sarcomere paced at CL= 300 ms with stable Ca cycling (*u*=1.5 ms^-1^). Initial conditions are chosen so that sarcomeres above (below) the 12th sarcomere start with opposite alternans phase.

To understand the underlying mechanism for node formation and drift it is necessary to study the bidirectional coupling between voltage and subcellular Ca. To address this issue we will first analyze the response of subcellular Ca to an AP clamp. This scenario is particularly relevant in tissue since the voltage driving a cell is determined by the averaged voltage from a large population of cells. Thus, on the length scale of a single cell, the membrane voltage is effectively clamped. In Figure 4A-C we simulate the dynamics of a Ca alternans node in response to an AP clamp that is periodic (A), alternates in a LSLS (B), and SLSL sequence (C). In this simulation, we have chosen initial conditions such that a node is formed at the 12^*th*^ sarcomere. To accomplish this we have paced a single sarcomere to steady state at a cycle length of 300 ms and then recorded the Ca concentration at the end of alternate beats. We then use the initial conditions above (below) the 12th sarcomere using Ca concentration values at the end of the large (small) beats. Therefore, as the system is paced further the regions with different initial conditions evolve with opposite alternans phase. The AP clamp that we apply is generated by using an AP alternans waveform recorded from a single sarcomere simulation at a cycle length of 300 ms with stable Ca cycling (*u*=1.5 ms^-1^). We observe that when the AP clamp is periodic the node remains pinned to the location where it is formed. Note that Ca alternans persists even in the presence of an AP clamp since our system parameters are chosen such that the alternans instability is due to Ca cycling. However, if the AP alternates either LSLS or SLSL then the node drifts towards the top or bottom of the cell and eventually whole Ca transients become lsls with LSLS or slsl with SLSL. Our results are consistent with the idea that nodes always drift in a direction such that the final steady state pattern of Ca transient alternans is in phase with the applied AP clamp. 

### Synchronization of CaT alternans in several coupled cells

**Figure 5:**
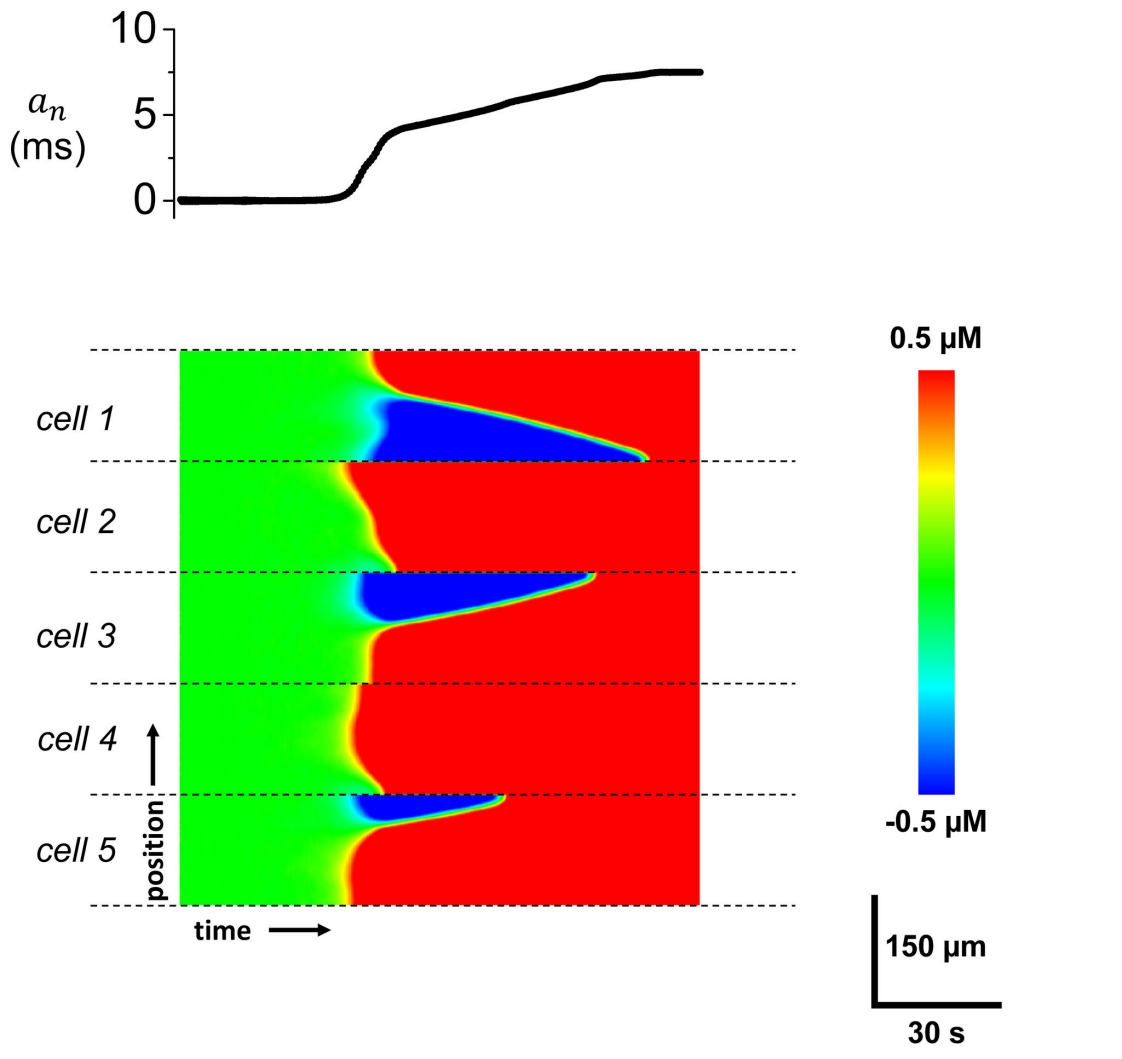
Development and synchronization of alternans within 5 coupled cells. The amplitude of APD alternans (top) and a space-time plot of subcellular Ca (bottom). Dashed lines indicate cell boundaries.

In this section, we will investigate the spatiotemporal dynamics of *V*_*m*_ and Ca alternans in a multi-cell setting. As a starting point, we will consider a system of five cells that are electrically coupled via gap junctions. Here, we assume voltage is spatially uniform since the equilibration time of voltage is effectively instantaneous at this length scale. In these simulations, we set Ca diffusion between cells to be zero since experimental studies indicate that Ca permeation across gap junctions is small, and is unlikely to influence the phase relationship Ca alternans. In Figure 5 we show the time course of APD alternans amplitude along with a space-time plot of subcellular Ca within the 5 coupled cells. We observe that after 40s spatially discordant patterns of Ca transient alternans develops in some cells, with out-of-phase regions of red and blue separated by a node. Now, as the APD alternans amplitude increases these nodes drift so that at steady state, the 5 cell system is synchronized with the phase (red) that is concordant with the APD signal. Thus, in a multi-cell system spatially discordant alternans, which form early, coarsen and evolve towards a steady state phase that is electromechanically concordant with the APD driving the system. However, we note that in some cases alternans phase of a single cell can be "trapped" out-of-phase with the APD signal. This scenario occurs when a cell is spatially synchronized (no node is formed) and is also electromechanically out-of-phase with the APD waveform. In these cases, we find that if the amplitude of APD alternans is smaller than a critical value then the voltage waveform does not reverse the Ca alternans phase (see Online Supplement text and Figures S3-S4 for a detailed description of this scenario). However, we find that these cases are rare and do not play an important role in the spatial distribution of alternans on the tissue scale. 

### Spatiotemporal dynamics of Ca and voltage alternans on a cable

**Figure 6:**
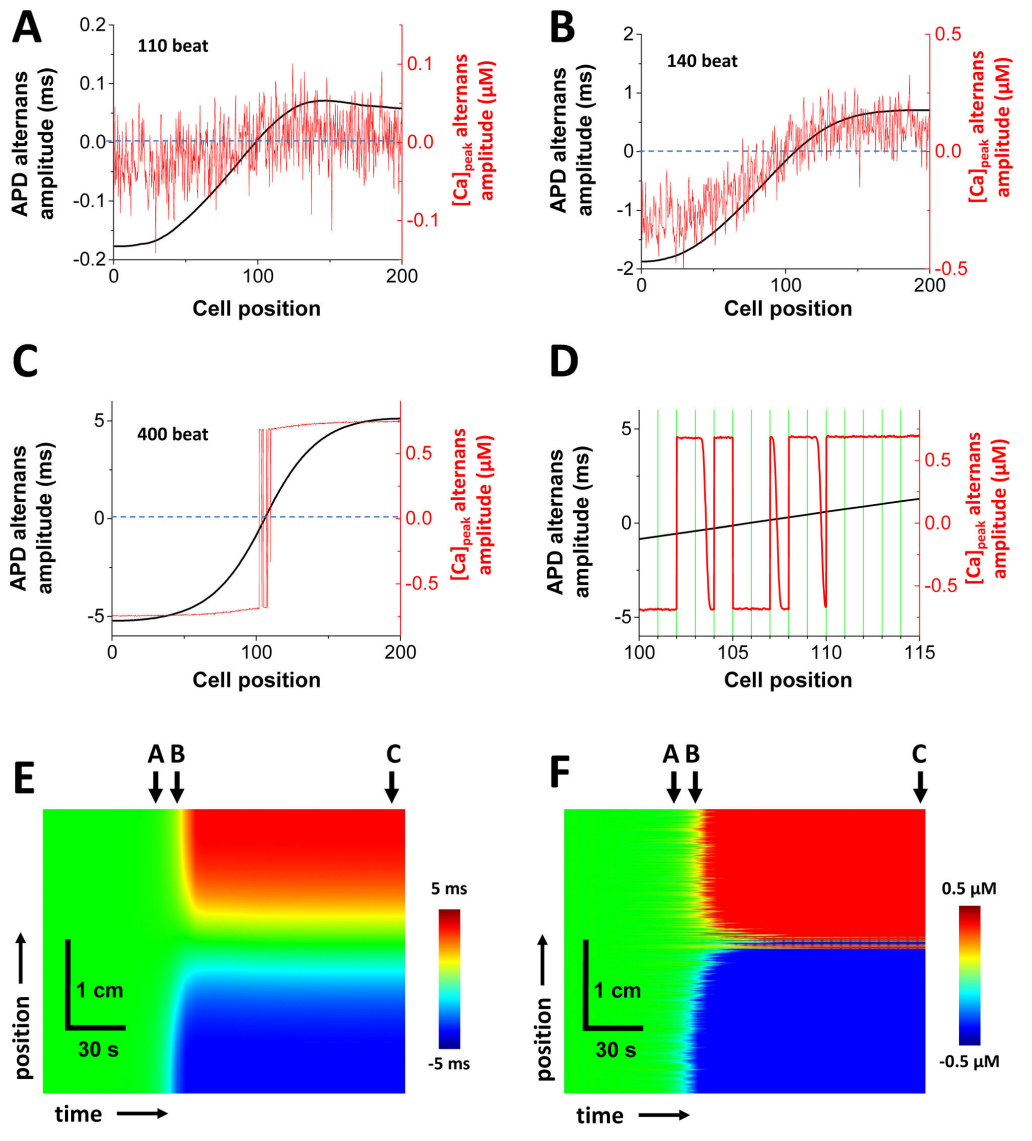
Development of SDA in a cable of 200 cells (3cm). The profiles of the amplitudes of APD and CaT alternans at (A) beat 110, (B) beat 140, and (C) beat 400. (D) Details of the profiles at 400 beat between 100 and 115 cells. Green lines indicate cell boundaries. Since the amplitude of APD alternans is small, it will take time to synchronize these subcellular SDA. Space-time plots of the APD alternans amplitude (E) and the CaT alternans amplitude (F).

In this section, we will analyze the spatiotemporal dynamics of voltage and Ca in a cable of 200 coupled cells. As in the single cell simulations we pace all cells on our cable simultaneously to steady state at CL=600 ms, and then decrease the pacing rate to CL=300 ms. In Figures (6A-C) we show the spatial distribution of Ca and APD alternans amplitude after beats 110,140, and 400. During the first 110 beats Ca alternans amplitude is small and forms a highly disordered pattern of out-of-phase regions. These regions occur on a length scale of a single cell and are separated by Ca alternans nodes within cells, or at cell boundaries. These fine scale patterns gradually coarsen as both Ca and APD alternans amplitude increases. At the 400th beat (close to the steady state) APD alternans forms a discordant pattern with one node on the cable (Figure 6C). Note that the Ca alternans amplitude is similar to APD but with multiple nodes concentrated near the node where APD alternans amplitude is small (Figure 6D). The space-time evolution of alternans is shown in Figure 6E-F, showing the slow gradual evolution of SDA on the cable. Repeated simulations of the cable, using identical system parameters, show that the final steady state pattern is similar but where the precise location and shape of the SDA pattern is random (Figure S1). In general we observe that in the vicinity of a region of large APD alternans the Ca transient alternans are synchronized, although there can be regions of cells which are trapped out-of-phase with the local APD. However, in regions near the nodes, where the APD amplitude is small, Ca alternans can reverse phase over a subcellular scale. In Figure 6F we show the spatial profile of subcellular Ca near the APD node for the simulation shown in Figure 6C. Here, we observe 9 nodes of which 6 occur on a cell boundary, and 3 inside the cell. We find that these subcellular nodes drift to synchronize with the APD alternans phase, but they do so at an extremely slow rate. The reason for this is that node motion is driven by the local APD alternans, which in this case is very small. However, we note that after several hundred beats the nodes eventually reach the cell boundaries. Thus, at steady state all nodes on the cable occur only at cell boundaries. This motion is very slow since the APD alternans amplitude is small near the node. 

### Spatiotemporal dynamics of Ca and voltage alternans on 2D tissue

**Figure 7:**
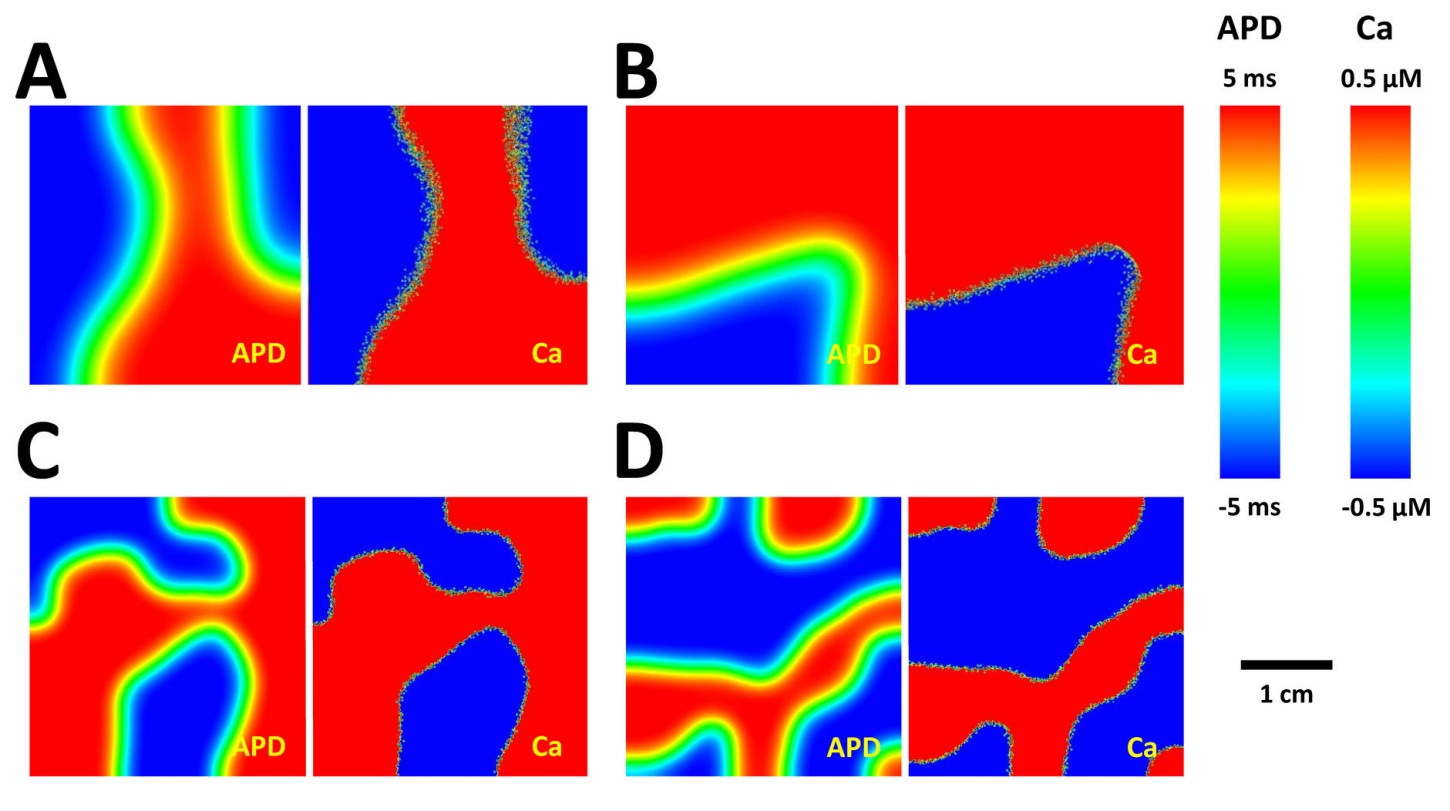
Steady state patterns of APD and CaT alternans in 2D tissue. Two independent simulations with *D*_*v*_=2.5×10^-3^ cm^2^/ms (A-B), and *D*_*v*_=6.25×10^-4^ cm^2^/ms (C-D).

We have applied our numerical simulation approach to model the distribution of alternans in 2D cardiac tissue. In Figure (7A-D) we show four independent simulations where a square cardiac tissue of 200 by 200 cells is paced. Here, all cells are paced simultaneously so that SDA cannot be formed via a steep CV restitution [12,13,33]. As in the 1D scenario, we find that the final steady state APD alternans pattern always displays SDA. Likewise, the steady state Ca alternans amplitude shows a similar pattern on the tissue scale (cm) but can reverse phase multiple times at cells in the vicinity of APD nodal lines. We note that the position of nodal lines appears disordered and varies substantially between each simulation run. 

### Cable paced from one end

**Figure 8:**
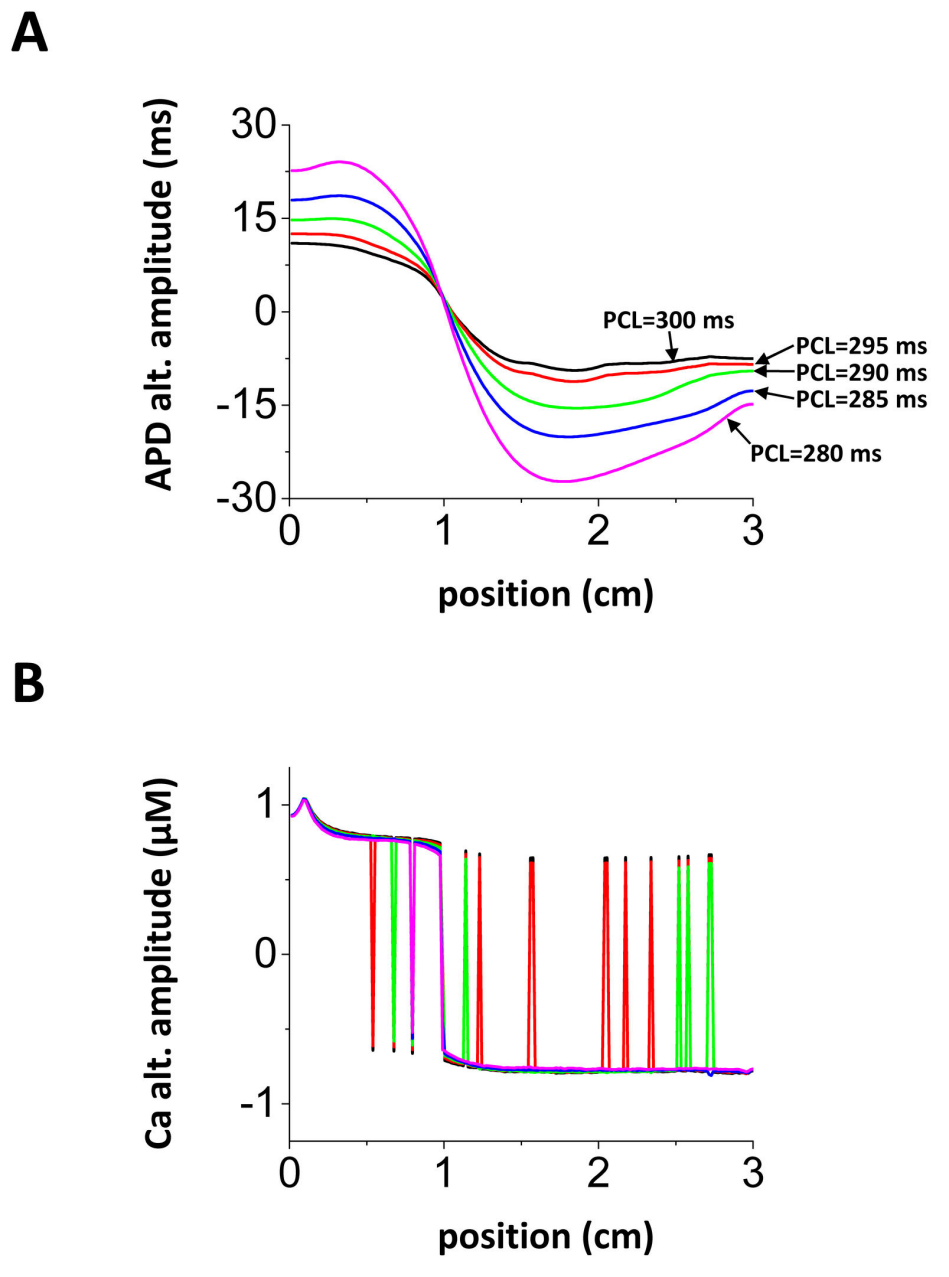
Tissue paced from the end. Steady state APD (A) and Ca (B) alternans amplitude when PCL is changed from 300 ms to 280 ms. The leftmost 5 cell (at 0 cm) are paced in the 1D cable.

Cardiac tissue is typically paced at a local site from which the excitation propagates and excites the rest of the tissue. Therefore, the time of arrival of an excitation can vary spatially due to interactions between local APD changes and the CV. Watanabe et al.[10] showed that if a cable is paced rapidly at one end, at a rate which engages the steep portion of the CV restitution curve, then SDA can form via a purely dynamical mechanism. In that study it was noted that nodes formed due to APD and CV restitution and moved towards the pacing site in response to an increase in pacing rate. Here, we will investigate the relationship between node position and pacing rate in the scenario where SDA is due to the fluctuation induced mechanism proposed here. In Figure 8 we show the amplitude of APD alternans across a 3 cm cable that is paced at 5 leftmost cells at one end. We gradually decreased the pacing interval from 300 to 280 ms and found that the site of the node did not change in response to the change in CL. This result is in sharp contrast to the CV induced mechanism for SDA where the node always moved towards the pacing site in response to faster pacing. Thus, it may be possible to use the tissue response to a dynamic pacing protocol to identify the potential mechanisms for SDA. 

### Formation of Wave break

**Figure 9:**
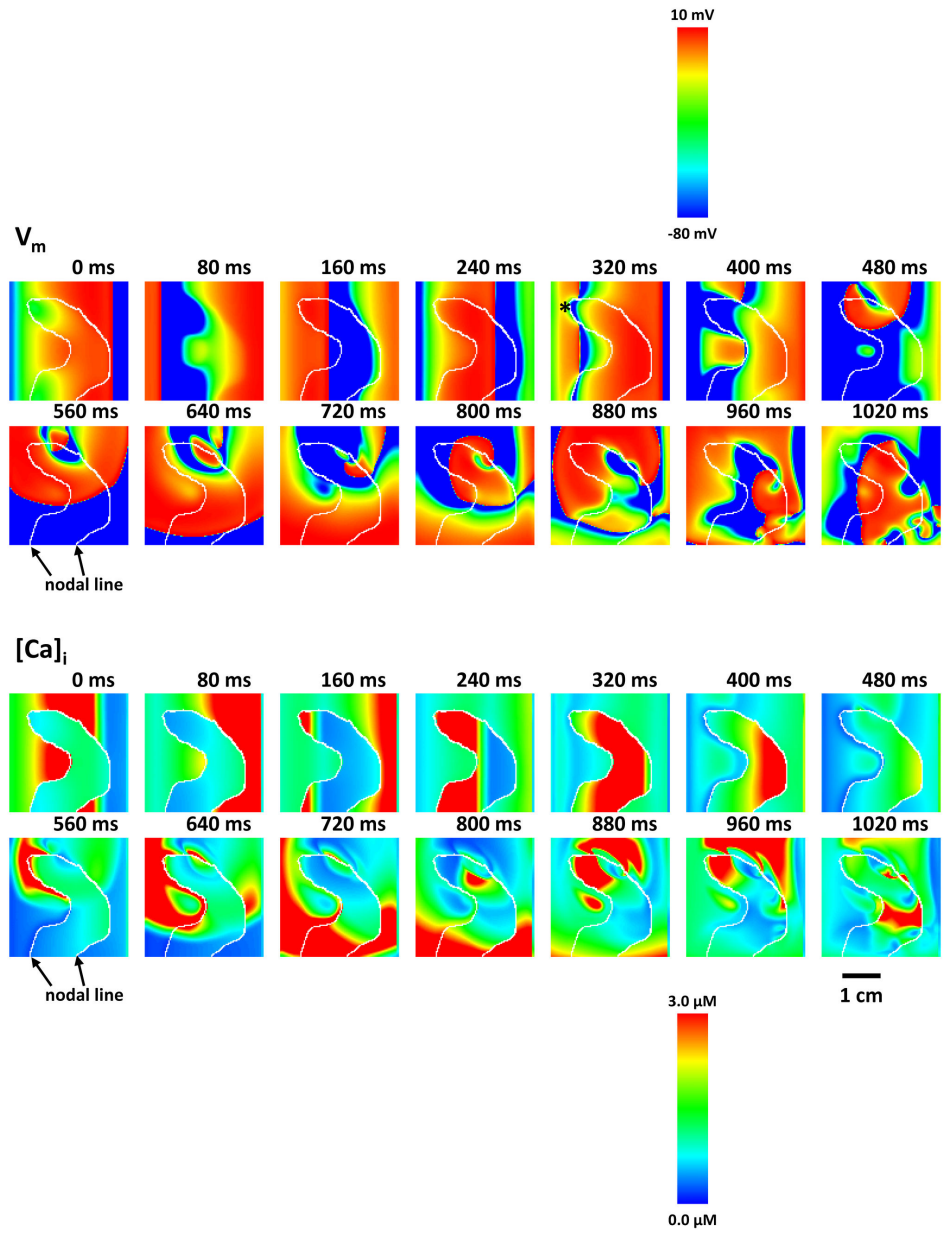
Initiation of ventricular tachycardia and fibrillation. 14 snapshots of the membrane voltage and [Ca]_i_ in 3 cm x 3 cm tissue. Tissue paced from the left edge. White lines indicate nodal lines, which separate opposite phase of alternans. At 320 ms S2 wave hit the S1 waveback (induced by *). Then a spiral wave (i.e. ventricular tachycardia) is initiated (460-960 ms). The spiral wave spontaneously brakes up at 1040 ms.

SDA is arrhythmogenic since it leads to the formation of APD heterogeneity, which can form a substrate for wave break and reentry. To confirm this relationship in our model we induce steady state SDA at a fixed pacing cycle length S1, and then deliver a pulse at a shorter interval S2. Our aim is to see if the SDA pattern formed at rate S1 is sufficient to induce wave break of the S2 pulse. In Figure 9 we show snapshots of AP propagation of an S2=200 ms wavefront following steady state pacing at S1=280 ms. The white line indicates the Ca nodal line formed at the steady state pacing rate. In this example we observe wavebreak of the S2 wavefront at time *t*=320 ms, which then evolved into a reentrant pattern (see online Movie S1). A close examination reveals that wavebreak occurred due to nonuniform conduction near nodal lines that where parallel to the direction of propagation (see snapshot at *t*=320 ms). These sites were prone to wavebreak since those are the regions with the maximum change in CV across the wavefront. In repeated simulation runs, we observed that wavebreak always occurred at nodal lines, and more precisely in regions where the nodal lines were perpendicular to the wavefront. Thus, the disordered pattern of SDA, formed via the mechanism proposed here, is sufficient to induce wavebreak. 

## Discussion

### Mechanism of SDA formation

**Figure 10:**
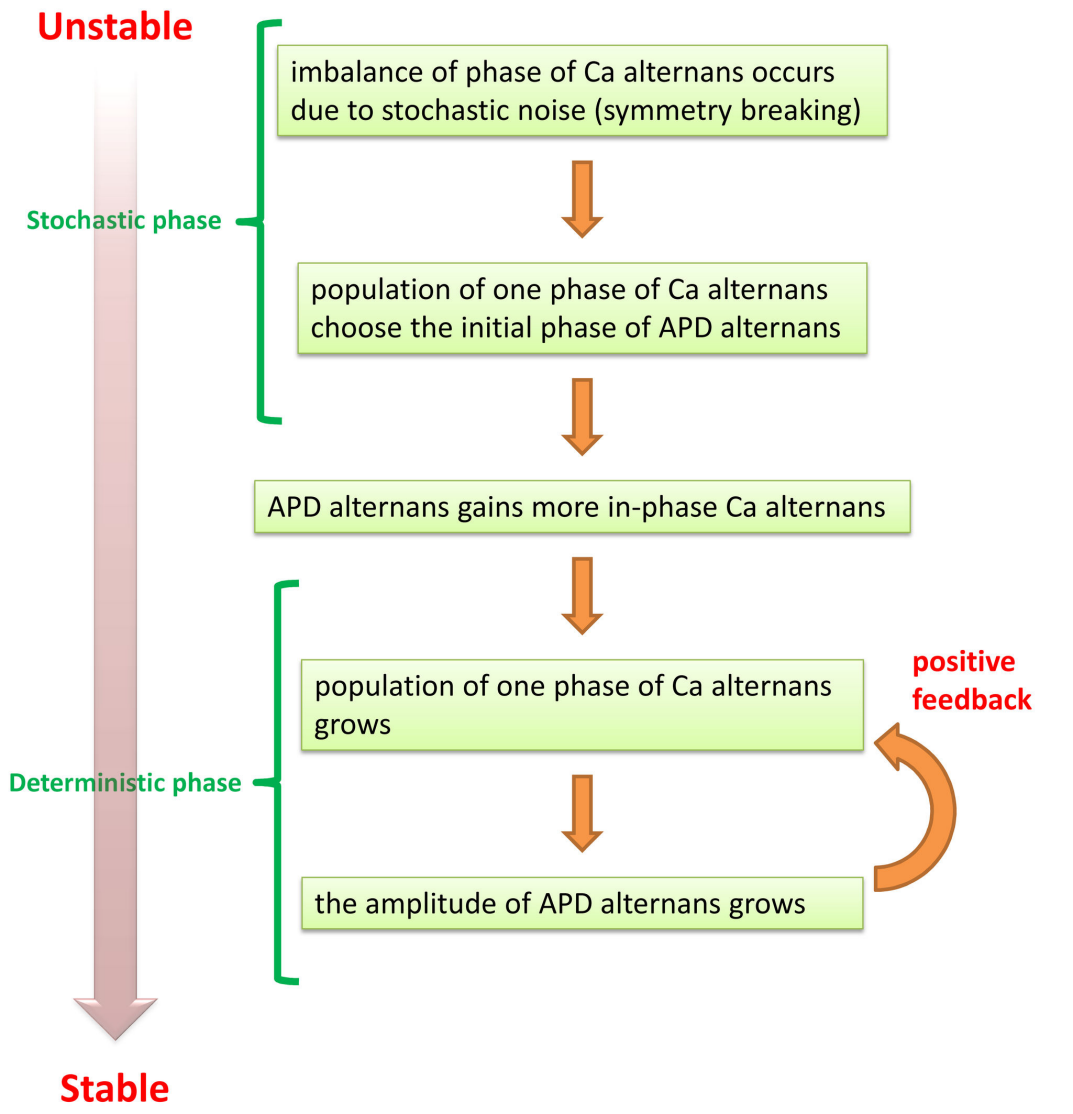
Summary of the mechanism for the formation of SDA.

In this paper, we present a novel mechanism for the formation of SDA in cardiac tissue. The main requirements for this mechanism to apply are that: (i) Cardiac alternans is due to an instability in Ca cycling dynamics. (ii) The coupling between Ca and voltage is positive (electromechanically concordant). (iii) Alternans develops due to Ca accumulation, which occurs over many beats. Under these conditions, we demonstrate that rapidly paced cardiac tissue will form steady state SDA. To understand the mechanism for this SDA formation we have applied a multi-scale computational approach that describes voltage and Ca at the scale of a single sarcomere, whole cell, multi-cell, and tissue. Our computational studies reveal that SDA is formed gradually due to a coarsening process dictated by the bi-directional coupling between subcellular Ca and voltage. During the first few beats Ca alternans amplitude is small and subcellular regions in tissue evolve to an alternans phase that is dictated by fluctuations in Ca cycling. Thus, spatially discordant Ca transient alternans form at the subcellular scale leading to a highly heterogeneous Ca transient distribution in a tissue of cells. In this regime, APD alternans amplitude is small and is not sufficient to synchronize the out-of-phase regions of Ca alternans. However, as Ca accumulates in the SR and the amplitude of Ca alternans increases, then APD alternans amplitude also increases and begins to synchronize Ca over a length scale dictated by the electrotonic coupling in tissue. As Ca alternans are synchronized in multiple cells, the amplitude of APD alternans also grows. This positive feedback process is responsible for synchronizing alternans in multiple cells in tissue. In Figure 10, we summarize the mechanism for the partial synchronization. In large tissue, this coarsening process typically leads to steady state patterns of SDA since distant regions of tissue are weakly coupled and can evolve towards opposite alternans phase.

### The scale of pattern

The slow coarsening of APD and Ca alternans amplitude is dictated by the effective spread of voltage and Ca in cardiac tissue. Recall, that Ca diffuses in the cell with a diffusion coefficient roughly *D*_*Ca*_~3×10^-6^ cm^2^/s [34,35], while the effective diffusion coefficient of voltage in cardiac tissue is *D*_*V*_~1 cm^2^/s. During rapid pacing the CL is typically ~300 ms so that the effective distance Ca diffuses on this time scale is

$$\xi_{Ca}\text{\textasciitilde{}}\sqrt{D_{Ca}\cdot CL}\text{\textasciitilde{}}0.01\text{ mm}$$ (5)

which is roughly the width of a few sarcomeres. Thus, at each beat the subcellular Ca concentration is smoothed over a length scale given by ξ_*Ca*_. Thus, discordant Ca alternans patterns can vary only over scales larger than ξ_*Ca*_, which sets a minimum distance between Ca alternans nodes. On the other hand, APD alternans are smoothed over a much larger scale given by 

$$\xi_{V}\text{\textasciitilde{}}\sqrt{D_{V}\cdot CL}\text{\textasciitilde{}}5\text{ mm}$$ (6)

which sets the minimum spacing between APD alternans nodes. Furthermore, the length scales ξ_*Ca*_ and ξ_*V*_ determine the effective interface width separating regions of out-of-phase Ca and APD alternans respectively. The vast difference in these length scales determine essential features of the spatiotemporal coarsening dynamics of alternans. The key effect is that APD alternans in a region of tissue is dictated by the distribution of Ca alternans from a population of cells within a radius ~ ξ_*V*_. On the other hand, the resulting APD alternans in that region dictates the subcellular Ca distribution since Ca alternans nodes drift in response to the voltage driving each cell. The interplay between these length scales can be observed in the 2D simulations shown in Figure 7. We note that the interface separating out-of-phase APD alternans occurs on a scale comparable to ξ_*V*_, while Ca alternans varies on a much finer length scale. However, we observe these fine scale Ca alternans patterns only close to APD nodal lines, where the amplitude of APD is not sufficient to synchronize Ca. Here, we emphasize that the length scales ξ_*V*_ and ξ_*Ca*_ only determine the minimum length scales over which APD and Ca alternans patterns can vary. The precise steady state spatial distribution of Ca and APD nodal lines is determined by the details of the coarsening dynamics, which occurs over many beats. 

### Stochastic and deterministic factors for the formation of SDA

In this study, we have shown that subcellular fluctuations of Ca cycling dynamics can play an important role in SDA formation. During the early stages of pacing, when Ca alternans amplitude is small, fluctuations play a crucial role to choose the alternans phase. Fluctuations can arise from a variety of factors such as the number of Ca sparks recruited at each beat, Ca concentration fluctuations within subcellular compartments, or the inherent stochasticity of ion channels controlling the flow of Ca in the cell. In this study, we opted for a minimal model which accounted only for the finite number of SERCA pumps within a sarcomere. Therefore, we expect that in a real cell subcellular fluctuations will be even larger than the case considered here. In that scenario the initial spatial distribution of Ca alternans amplitude is likely to be even more disordered, and spatially discordant alternans should form earlier in the pacing sequence. However, once the alternans phase is chosen and the APD and Ca amplitude grow towards their steady state values, then local fluctuations do not influence the pattern forming process. Thus, the location of nodes will be crucially dependent on Ca cycling fluctuations at the early stage of pacing, but the steady state average node spacing will be largely independent of the initial underlying fluctuations. To confirm the insensitivity of our result to the source of noise we have also simulated fluctuations of SR Ca. In this approach we introduce a noise term in the SR Ca release current so that this current fluctuates up to 10*%* of its average value. In Figure S2 (online supplement) we show the spatiotemporal evolution of SDA and confirm that the spatiotemporal dynamics is similar to the case when the noise was due to SERCA current fluctuations. Thus, the shape and location of nodes are disordered and vary substantially from sample to sample, but large scale features, such as the average node spacing, are essentially the same. 

An essential requirement for SDA to form is that alternans amplitude grows slowly over many beats following a change in CL. This is crucial since the initial discordant pattern was formed by fluctuations which broke the phase symmetry of the initially stable state. However, a sudden decrease in CL favors a SLSL alternans sequence since a "small" Ca transient will follow the first paced beat at the reduced CL i.e. there will be less Ca release on that first beat, which will lead to a larger beat on the next. Therefore, a sudden change in CL breaks the symmetry between opposite phases and forces the cell into one synchronized phase. The slow Ca accumulation in our model is essential to wash out this initial perturbation and return the system to the stable state, after which small fluctuations will determine the alternans phase. Thus, it is important to stress that the mechanism proposed here will be sensitive to the pacing protocol. In particular, in the scenario where the system is paced such that the initial SR load is close to the threshold for alternans, then a change in CL will force the system to remain in the phase dictated by the first beat. In this case, the CL perturbation will chose the phase of the system and we will expect only concordant alternans to occur in tissue. However, many experiments have shown that Ca accumulation is a slow process and that a sudden change in CL is likely to induce a stable transient before alternans onset. For example, Diaz et al. [36] investigated the growth of alternans in rat myocytes following depletion of the SR due to caffeine, and found that Ca overload occurred after roughly 50s of pacing. Thus, in this case, Ca accumulation is slow and the initial perturbation is likely to be washed out after several beats of pacing. In this case, fluctuations will dictate the coarsening process and the pattern forming mechanism predicted here should apply. 

### Required conditions for this pattern forming mechanism

A necessary condition for the proposed mechanism to apply is that alternans at the cellular scale are due to an instability of Ca cycling. The critical role of Ca in the formation of alternans is now well established in the literature. In particular, Chudin et al[9]. demonstrated the presence of Ca transient alternans in rabbit cells that are rapidly paced with a periodic AP clamp. This result demonstrates convincingly that alternans, in this particular case, is due to an instability in Ca cycling dynamics, rather than a voltage instability driven by a steep APD restitution slope. Furthermore, it is now well known that in many diseased states mutations of the RyR channels are directly linked to the formation of Ca transient alternans[37,38]. These results demonstrate that alternans can originate from a disruption of a specific molecular target in the Ca cycling machinery. Our findings here predict that rapidly paced cardiac tissue, under these diseased conditions, will be prone to the patterns of SDA demonstrated here. 

A second important requirement for the proposed mechanism to apply is that Ca and APD should have "positive coupling" as defined by Shiferaw et al[8]. In this case a large (small) Ca transient corresponds to a long (short) APD at steady state i.e. that alternans are electromechanically concordant. This phase relationship is dictated by the coupling between the Ca transient and the APD via the combined effect of the L-type Ca current (*I*_*Ca*_) and the sodium-Ca exchanger (*I*_*NaCa*_). In effect, Ca and APD are electromechanically concordant if the increase of inward currents due to Ca extrusion via *I*_*NaCa*_ is larger than the decrease due to Ca induced inactivation of *I*_*Ca*_. Experimental observations of simultaneous recordings of voltage and Ca reveal that this is indeed the case under a wide variety of conditions [30,39,40]. In fact, the vast majority of experiments have found that Ca and APD alternans are electromechanically concordant [7,9,31,32]. Hence, this requirement for SDA formation is likely to apply in a wide variety of cases in which Ca transient and APD alternans are observed [9,30,39,40]. 

### Distinct features from other SDA mechanisms

The formation of SDA in tissue has been studied by a number of investigators using both experimental and theoretical approaches. Watanabe[10] and Qu[11] first identified a mechanism for the formation of SDA, which occurred when cardiac tissue is paced at a site, and where the conduction velocity (CV) varies substantially with the diastolic interval (DI). Here, SDA occurs due to variations in the timing of excitation at cells away from the pacing site. In this case, these authors showed that nodal lines were oriented radially with respect to the pacing site, and moved towards the pacing site as the CL was decreased. However, these studies strictly applied to the case where alternans were due to a steep APD restitution. Recently, Skarsdal et al [15] showed that in the case when Ca cycling was the driver for alternans then a similar phenomenon occurs with the key difference that nodes were unidirectionally pinned, so that nodes could move towards the pacing site but not away. However, in both cases the underlying mechanism for SDA could be identified by the orientation of nodes around the pacing site, and also the motion of nodes towards the pacing site in response to a decrease in CL. In the mechanism reported here steady state SDA is essentially static and nodes do not move towards the pacing site in response to a change in CL. Thus, the response of nodes to changes in CL could serve as an indicator to distinguish the underlying mechanism for SDA formation. In an experimental study Hayashi et al. measured the motion of nodal lines in the intact rabbit heart in response to changes in CL[33]. These authors found a variety of complex nodal patterns with different responses to CL changes. In some instances nodes where found to move in response to change in CL, consistent with a CV induced mechanism. However, several cases were reported where nodal lines were not oriented radially with respect to the pacing site, and also did not move in response to changes in CL[41]. It is possible that in these cases the formation of SDA is due to the fluctuation mechanism proposed here. Interestingly, we found that the disordered pattern of nodal lines that are formed via this mechanism have a higher likelihood of inducing wave break. This is because nodal lines oriented perpendicular to the wave front induce greater conduction heterogeneities than when the lines are parallel. This results suggests that the disordered SDA patterns formed via this mechanism are more arrhythmogenic than in the case due to CV restitution, where the induced nodal lines are always oriented parallel to the wavefront. 

## Conclusions

In this paper, we have explored a novel mechanism for the formation of SDA in cardiac tissue. This mechanism may have considerable relevance to arrhythmias under diseased conditions in which Ca cycling is abnormal. In fact, numerous experimental studies of Ca cycling abnormalities find that alternans development is a common occurrence, especially under rapid pacing or Ca overload conditions[21]. For instance, cardiac cells in heart failure are known to have abnormal Ca cycling and exhibit alternans at rapid pacing rates[20]. Also, gene based studies of CPVT find that mice with an RyR mutation exhibit alternans at rapid pacing rates[42,43]. Furthermore, in the clinical setting a change in cyle length from slow to fast occurs frequently under a variety of physiologically relevant conditions. For example, during exercise the heart rate increases rapidly and will lead to slow ion accumulation as the heart adjusts to the new pacing rate [44]. In the scenario where the cardiac cells are prone to Ca cycling alternans, then the mechanism for the formation of SDA proposed here, is likely to apply. This result may explain the heightened propensity for arrhythmias in these tissues, under rapid pacing, since the likelihood of wave break and reentry is substantially increased by the gradients of refractoriness induced by SDA. 

## Supporting Information

Figure S1 **The profiles of the amplitudes of APD and CaT alternans in a cable of 200 cells (3cm) at 400 beats.** Three examples (A)~(C). The parameters and initial conditions are the same as the simulation in Figure 6. Stochastic noise at the beginning of the development of alternans creates different patterns.
(TIFF)

Figure S2 **Development of SDA in a cable of 200 cells (3cm) due to fluctuation in SR Ca releases.** The profiles of the amplitudes of APD and CaT alternans at the steady state.
(TIFF)

Figure S3 **The amplitudes of APD and CaT alternans at the steady state when developed lsls CaT alternans is paced with an AP clamp waveform in a SLSL sequence.**
(TIFF)

Figure S4 **Development and synchronization of alternans within the 5 coupled cells.** One cell (cell 1) is trapped in out-of phase CaT alternans. The amplitude of APD alternans and a space-time plot of subcellular Ca.
(TIF)

Movie S1 **The movie of Figure 9.** See Figure 9 for a detailed description of this movie.
(MP4)
